# Effects of a short and intensive transcranial direct current stimulation treatment in children and adolescents with developmental dyslexia: A crossover clinical trial

**DOI:** 10.3389/fpsyg.2022.986242

**Published:** 2022-09-09

**Authors:** Andrea Battisti, Giulia Lazzaro, Floriana Costanzo, Cristiana Varuzza, Serena Rossi, Stefano Vicari, Deny Menghini

**Affiliations:** ^1^Child and Adolescent Neuropsychiatry Unit, Department of Neuroscience, Bambino Gesù Children’s Hospital, IRCCS, Rome, Italy; ^2^Department of Human Science, LUMSA University, Rome, Italy; ^3^Department of Life Science and Public Health, Catholic University of the Sacred Heart, Rome, Italy

**Keywords:** specific learning disorders, non-invasive brain stimulation, interventions, reading, neuroplasticity

## Abstract

Developmental Dyslexia (DD) significantly interferes with children’s academic, personal, social, and emotional functioning. Nevertheless, therapeutic options need to be further validated and tested in randomized controlled clinical trials. The use of transcranial direct current stimulation (tDCS) has been gaining ground in recent years as a new intervention option for DD. However, there are still open questions regarding the most suitable tDCS protocol for young people with DD. The current crossover study tested the effectiveness of a short and intensive tDCS protocol, including the long-term effects, as well as the influence of age and neuropsychological processes at baseline on reading improvements. Twenty-four children and adolescents with DD were randomly assigned to receive active tDCS during the first slot and sham tDCS during the second slot or vice versa. Five consecutive daily sessions of left anodal/right cathodal tDCS set at 1 mA for 20 min were administered over the parieto-occipital regions. Reading measures (text, high frequency word, low frequency word, and non-word lists) and neuropsychological measures (visual-spatial and verbal working memory, phoneme blending, and rapid automatized naming tasks) were collected before, immediately after, 1 week and 1 month later the treatment. Our results showed that only the active tDCS condition improved non-word reading speed immediately after and 1 month later the end of the treatment compared with baseline. In addition, the improvement in non-word reading speed was significantly correlated with age and with neuropsychological measures (verbal working memory and phoneme blending) at baseline but only in the active tDCS condition. The current crossover study contributed to enforce previous effects of tDCS, including long-term effects, on non-word reading speed and to understand the effect of age and neuropsychological processes on reading outcomes. Our findings showed that tDCS could be a low-cost and easy-to-implement treatment option with long-term effects for children and adolescents with DD.

## Introduction

Among reading difficulties, Developmental Dyslexia (DD) is a severe and long-lasting impairment of reading skills acquisition, specifically characterized by inaccurate and/or non-fluent word recognition and poor spelling and decoding abilities, in absence of neurological, sensorial, and cognitive deficits or educational under-exposure (American Psychiatric Association [APA], 2013). With an estimated prevalence of 7% (Yang et al., 2022), DD consists of a neurobiological-based disorder that covers about 80% of all learning disabilities (Mee Bell et al., 2003; Shaywitz et al., 2007) and is distinguished by difficulties in reading comprehension at higher levels.

Although several interpretative theories of DD have been proposed over the years (for a review, see Peterson and Pennington, 2012), extensive evidence converges to consider DD as a multifactorial disorder with heterogeneous manifestations (Menghini et al., 2010). Accordingly, DD has been associated with neurofunctional abnormalities of a broad cerebral network in the left posterior hemisphere: a well-documented under activation of left temporo-parietal regions – mainly involved in lexical access and phonological processing – and left occipito-temporal regions – mainly involved in the fast word recognition – compared to typical readers (for a review, see Richlan, 2020). Moreover, parieto-occipital regions have been shown to be implicated in whole-word representations (Graves et al., 2008), in reading morphologically complex words (Zweig and Pylkkänen, 2009) and during the comprehension of complex linguistic units (Jobard et al., 2007).

Multiple neurocognitive domains were found to be impaired in children and adolescents with DD. Several studies have shown that children with DD often have difficulties in phonological and non-phonological skills, such as in working memory (Gathercole et al., 2006; Beneventi et al., 2010; Wolf et al., 2010; Menghini et al., 2011), auditory and visual selective attention (Hari and Renvall, 2001; Bosse et al., 2007; Roach and Hogben, 2007; Facoetti et al., 2010; Lallier et al., 2010; Franceschini et al., 2012; Zorzi et al., 2012), executive functions (Willcutt et al., 2005; Shanahan et al., 2006; Varvara et al., 2014), automatization of sub-skills (Nicolson and Fawcett, 1990; Nicolson et al., 2001), and implicit and procedural learning (Vicari et al., 2003, Menghini et al., 2006). There is also evidence for difficulties in motion perception, as supported by the magnocellular deficit theory, and for visual-perceptual impairments (Galaburda and Livingstone, 1993; Kevan and Pammer, 2008, 2009; Menghini et al., 2010; Boets et al., 2011; Gori et al., 2014).

Given its functional impairment and impact on learning, DD is recognized as a risk factor for reduced socio-economic outcomes (Carroll et al., 2005; Aro et al., 2019) and the onset of emotional-behavioral difficulties (Hendren et al., 2018; de Lima et al., 2020; Wang, 2021; Xiao et al., 2022). Although some treatments, especially those based on phonics, have shown some efficacy in improving reading skills in children and adolescents with DD (Galuschka et al., 2014; McArthur et al., 2018; Wanzek et al., 2018), there is still some variability in response and treatments are not effective for all children (Gabrieli, 2009; Toffalini et al., 2021). These reasons drive the need to provide further testing and validation of treatments in DD.

In this context, the use of non-invasive brain-based methods has been gaining ground in recent years as a new intervention option for children and adolescents with DD (Cancer and Antonietti, 2018). Among these non-invasive brain-based methods, transcranial direct current stimulation (tDCS) has been the most widely used technique to improve reading accuracy and speed in typical readers and readers with DD (for a review, see Turker and Hartwigsen, 2022), especially when combined with reading trainings (Finisguerra et al., 2019). tDCS is a safe and highly tolerated method (Buchanan et al., 2021) and involves the application of a direct, low current (usually 1–2 mA) to the scalp through two sponge electrodes (anode and cathode). It has been shown to induce persistent neural changes and modulate behavior (Nitsche and Paulus, 2000; Woods et al., 2016).

In children and adolescents with DD, several studies have demonstrated the beneficial effect of tDCS – stand-alone or in combination with reading training – on reading tasks, especially in non-word reading (efficiency, accuracy as well as speed), word reading fluency and word recognition speed, low-frequency word reading accuracy as well as text reading accuracy (for a review, see Turker and Hartwigsen, 2022).

Whereas, in children and adolescents with DD, the neurocognitive mechanisms modulated by tDCS and potentially associated with improvement in reading tasks have been investigated by only two studies. Specifically, Costanzo et al. (2016a) found that compared to baseline, a single session of left anodal/right cathodal tDCS on temporo-parietal regions as well as the reverse polarity montage significantly modulated neuropsychological processes (i.e., phoneme blending and verbal working memory) along with changes in reading. In addition, Lazzaro et al. (2021a) demonstrated that, compared with the reverse polarity montage, a single session of left anodal/right cathodal tDCS improved non-verbal neuropsychological processes (i.e., motion perception and modified attentional focusing) along with changes in reading.

However, although the results of non-invasive brain stimulation in DD are generally promising, randomized clinical trials (RCTs) are still few and have some methodological issues.

First, tDCS studies for the treatment of DD are characterized by small sample sizes with a maximum of 27 participants (Lazzaro et al., 2021b) and conducted mainly with between-subjects design.

Second, existing results are fundamentally heterogeneous (Costanzo et al., 2016b,^2019^; Rios et al., 2018) probably due to high inter-subject variability. Indeed, it has been widely recognized that the influence of stable factors (demographical, neuroanatomical, and genetical), or transient/contextual factors such as vigilance, hormonal activity, participant engagement or task predisposition can significantly produce heterogeneous results and alter the generalizability of findings observed in tDCS studies (for a review, see Vergallito et al., 2022).

One possibility to overcome these limitations is to design studies with a larger number of participants and/or apply a crossover design. In fact, the crossover study design was introduced in clinical research to obtain an effect estimate with the same level of accuracy as a between-subjects design, increasing statistical power even with a small number of participants (Senn, 2002; Chow and Liu, 2009; Wellek and Blettner, 2012), and suppressing the inter-subject variability (Jones and Kenward, 2014; Lim and In, 2021).

Third, the medium- and long-term effectiveness of tDCS studies in DD has been poorly explored, and limited to studies in which stimulation was combined with reading training (Costanzo et al., 2016b,^2019^; Lazzaro et al., 2021c; Mirahadi et al., 2022).

In this context, the current study represents the first RCT employing a crossover design to investigate the efficacy of a short and intensive multi-sessions stand-alone tDCS intervention in children and adolescents with DD. Further, to evaluate the after-effects of a stand-alone tDCS intervention, the present study aims to evaluate the persistence of observed results in the medium and long-term.

Furthermore, despite the extensive evidence regarding the implication of domain-general cognitive processes in the occurrence of DD (Menghini et al., 2010), neuropsychological processes related to reading improvement following tDCS have been poor explored (Costanzo et al., 2016a; Lazzaro et al., 2021a). To overcome this limitation, the current study aims to investigate neuropsychological measures related to reading (i.e., working memory, phoneme blending, and rapid automatized naming) to verify whether 5 days of tDCS can modulate these domain-general processes in addition to reading as well as whether these domain-general processes at baseline influence reading improvement following tDCS treatment.

We tested the effect of five consecutive daily sessions and the medium- (1 week later) and long- (1 month later) term effect of left anodal/right cathodal tDCS over parieto-occipital regions without reading training in 24 children and adolescents with DD. In addition to the documented strong effect of tDCS combined with concomitant training (Costanzo et al., 2016b,^2019^; Lazzaro et al., 2021c), the results of previous studies (Turker and Hartwigsen, 2022) and our preliminary results (Lazzaro et al., 2021b) introduced the possibility of also considering short and intensive tDCS treatment without concomitant training in children and adolescents with DD. Furthermore, the choice to place bilateral tDCS on the parieto-occipital regions is based on evidence reporting their crucial role on whole-word representations (Graves et al., 2008), in reading morphologically complex words (Zweig and Pylkkänen, 2009) and during the comprehension of complex linguistic units (Jobard et al., 2007).

In light of this, we hypothesize that even short and intensive tDCS treatment can result in improved reading performance. The absence of a reading training associated with tDCS may help to understand the specific influence of five sessions of neurostimulation in inducing reading improvement and triggering medium- to long-term neuroplasticity processes. Furthermore, studying the effect of tDCS on neuropsychological measures that are most often impaired in DD may be a further step in understanding how reading may be modulated in relation to possible changes in domain-general processes.

Finally, starting from our previous results (Lazzaro et al., 2021c) and in accordance with studies indicating that pre-existing factors (e.g., age) may contribute to improvements after tDCS treatment (for a review, see Vergallito et al., 2022), we explored the association between age and reading improvement.

Indeed, individual factors influencing outcomes deriving from tDCS without reading training find merit to be investigated in order to improve the applicability of such treatment in children and adolescents with DD.

## Materials and methods

### Ethical committee

This study was performed under the Declaration of Helsinki and was approved by the local research ethics committee (process number 20120X002931). The study was registered at ClinicalTrials.gov (ID: NCT04244578).

### Participants

Participants were enrolled during the daily clinical activities of the Child and Adolescent Neuropsychiatry Unit at the Bambino Gesù Children’s Hospital (Rome).

The presence of DD was assessed by a team of expert clinicians, including a psychologist, a neuropsychiatrist, and a speech therapist according to the Diagnostic and Statistical Manual of Mental Disorders, Fifth Edition (DSM-5; American Psychiatric Association [APA], 2013), and using norm-referenced reading measures as text, word and non-word reading (Sartori et al., 2007; Stella and Tintoni, 2007; Cornoldi et al., 2010; Cornoldi and Colpo, 2012; Cornoldi and Candela, 2015). Participants met DD criteria when the accuracy or speed level was at least 1.5 standard deviations below the age mean. Children and adolescents with intellectual disability, a personal history of neurological diseases, a personal history of epilepsy or in a first-degree relative, other primary psychiatric diagnoses or comorbid neurodevelopmental disorder (e.g., attention deficit or hyperactivity disorder, depression, and anxiety), and had received treatment for DD in the 3 months prior to baseline screening were excluded. All participants had normal or corrected-to-normal vision.

After receiving or confirming the diagnosis of DD and ascertaining the inclusion criteria, the researcher asked the children and adolescents and their parents if they wished to participate in the study. Then, the objectives and design of the study, all related procedures and the effort required, and the basic principles of tDCS and its characteristics were presented in detail. The results of published studies over the years on the application of tDCS in children and adolescents with DD were also summarized to clarify the rationale of the proposed experiment. All participants and their parents agreed to participate in the study after the procedures had been fully explained and they gave written informed consent to the study.

**FIGURE 1:**
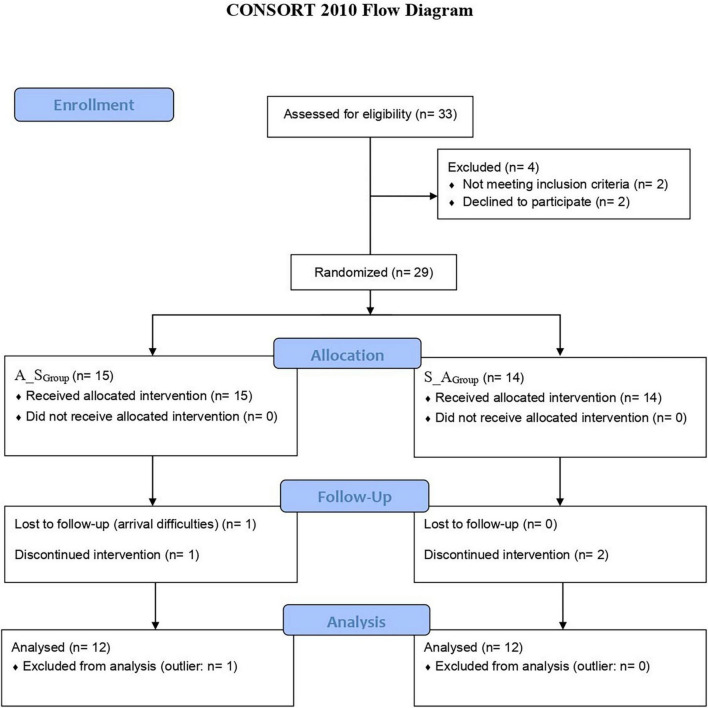
CONSORT flow diagram of the RCT.

As Figure 1 depicts (CONSORT flow diagram), 33 children and adolescents were screened for clinical eligibility, 29 of them were recruited and participated in the study. After the exclusion of 5 participants (1 outlier; 4 drops-out), a total sample of 24 native right-handed Italian-speaking children and adolescents with DD fully completed the crossover design and were considered for the study.

After clinical eligibility screening at baseline, recruited participants were randomized into two groups *via* minimal sufficient balancing method (to prevent imbalances in the baseline): A_S_Group_ [who received active tDCS during the first slot and sham tDCS during the second slot; age range: 9–17 years; females, F/males, M: 5/7; non-verbal IQ (nvIQ; Raven, 2008, 2009) range: 92–123] and S_A_Group_ (who received sham tDCS during the first slot and active tDCS during the second slot; age range: 10–18 years; F/M: 5/7; nvIQ range: 93–130).

Means (standard deviations – SDs) for chronological age, nvIQ, and *z*-scores of the norm-referenced reading measures at baseline are shown on Table 1. At baseline, the two groups did not differ for age (*p* = 0.10), nvIQ (*p* = 0.20), and clinical norm-referenced measures of reading: Text (Accuracy: *p* = 0.56; Speed: *p* = 0.86), Word (Accuracy: *p* = 0.92; Speed: *p* = 0.21), and Non-word (Accuracy: *p* = 0.63; Speed: *p* = 0.11).

**TABLE 1:** Means (SDs) of age, nvIQ, and *z*-scores of norm-referenced reading measures at baseline in the A_S_Group_ and S_A_Group_.

	A_S_Group_ *N* = 12	S_A_Group_ *N* = 12	*t*-Value	*p*-Value
Age	12.42 (2.45)	14.24 (2.68)	1.73	0.10
nvIQ	109.83 (10.61)	104.08 (10.79)	–1.32	0.20
**Text**				
Accuracy^a^	−2.34 (1.45)	−3.15 (4.53)	–0.59	0.56
Speed^b^	−2.37 (0.57)	−2.42 (0.80)	–0.18	0.86
**Word**				
Accuracy^a^	−2.26 (1.47)	−2.13 (4.30)	0.10	0.92
Speed^c^	−5.70 (2.92)	−4.08 (3.27)	1.28	0.21
**Non-word**				
Accuracy^a^	−1.36 (1.03)	−1.75 (2.53)	–0.49	0.63
Speed^c^	−3.88 (1.72)	−2.61 (2.06)	1.65	0.11

^a^Number of errors. ^b^Syllables/second. ^c^Seconds. nvIQ, non-verbal Intelligence Quotient.

### Sample size considerations

The sample size was calculated by *a priori* analysis in G*Power, version 3.1.9.7 (The G*Power Team, Düsseldorf, Germany).

To be conservative, we calculated the expected effect size (*f*) to medium/low and estimated it at 0.20.

With an estimated *f* = 0.20, α value = 0.05 (i.e., probability of false positives of 5%), β = 0.80 (i.e., at least 80% power), and a correlation among measures of 0.7, the sample size that was required for repeated-measures analysis of variance (RM ANOVA) with two conditions (Active vs. Sham) and four measurements (T0 vs. T1 vs. T2 vs. T3) was 22.

### Study design and procedures

A double blind, randomized, sham-controlled, crossover clinical trial was conducted.

Children and adolescents with DD underwent five consecutive daily sessions of active or sham tDCS (first slot, week 1). In the first slot, outcome measures were randomly administered at baseline (T0_1_), immediately after the end of the treatment (T1_1_), 1 week later (T2_1_), and 1 month later (T3_1_) by an investigator blinded to the stimulation conditions. After a 1 month washout (after the end of the T3_1_), children and adolescents who had received active tDCS during the first slot underwent five consecutive daily sessions of sham tDCS during the second slot, and vice versa. Similar to the first slot, in the second slot, outcome measures were administered randomly immediately before the start (T0_2_) and after the end of treatment (T1_2_), 1 week later (T2_2_) and 1 month later (T3_2_).

The study design and preliminary results – which include only participants who fully completed the first slot of either active or sham tDCS, assessment immediately post-treatment and 1 week later – were already presented in Lazzaro et al. (2021a).

**FIGURE 2:**
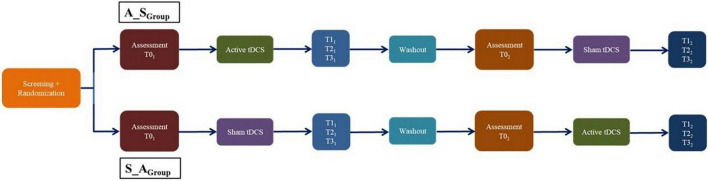
The crossover design.

Here, we will report the results of participants who fully underwent the crossover RCT, including treatment sessions and follow-ups (Figure 2).

All activities related to the study were conducted in a research laboratory at the Child and Adolescent Neuropsychiatry Unit of the Bambino Gesù Children’s Hospital in Rome.

### Outcome measures

To avoid the repetition effect, different versions of each task were considered, randomized between baseline and follow-up assessments (T0_1_, T1_1_, T2_1_, T3_1_, T0_2_, T1_2_, T2_2_, and T3_2_). To control for the effects of fatigue, task order was counterbalanced between assessments.

An extensive description of the proposed tasks were reported in Costanzo et al., 2016a,b, 2019 and Lazzaro et al., 2021a,b,c.

#### Reading tasks

Several reading tasks were presented, including: a text of more than 400 syllables (TEXT), a list of 20 high-frequency words (HF – 10 trisyllabic and 10 bisyllabic), a list of 20 low-frequency words (LF – 10 trisyllabic and 10 bisyllabic), and a list of 20 non-words (NW – 10 trisyllabic and 10 bisyllabic). A behavioral pre-test was conducted in children and adolescents with typical reading to select different versions of each set of stimuli (TEXT, HF, LF, and NW) that were equivalent in terms of difficulty in reading accuracy and speed (for more details, see Supplementary Table 1). Participants were asked to read aloud as rapid and accurate as possible.

For reading speed, the total time (in terms of seconds) taken to read HF, LF, and NW was measured. For TEXT, reading speed was calculated by dividing the total time (in terms of seconds) for reading completion by the total number of syllables spoken and multiplied by 100.

For reading accuracy, an error point was assigned in the presence of substitution, omission, addition of letters and in case of self-correction or hesitation during reading. For all reading tasks (TEXT, HF, LF, and NW), the percentage of accuracy was considered, calculated by multiplying the ratio of the number of correctly read stimuli to the total number of stimuli presented by 100.

#### Neuropsychological tasks

##### Working memory

Visual-spatial and verbal n-back tests were used to measure working memory.

The tests required participants to indicate whether the position of a colored box (visual-spatial n-back) moves to the same previous position or whether a pronounced letter (verbal n-back) matches the last pronounced letter (1-back). When the accuracy reached 80%, the difficulty increased and it was required to remember no longer the last position shown or the last letter pronounced, but the second-to-last (2-back), and, so on, the third-to-last (3-back), the fourth-to-last (4-back).

For both tests, an efficiency index (working-memory_Eff_) was calculated due to the highest n-back passed (when the percentage of accuracy value was above equal to or greater than 80%) followed by the percentage of accuracy of n-backs failed (when the percentage of accuracy value is <80%). For example, if a child achieves level 2-back but fails at level 3-back with an percentage of accuracy of 60%, the efficiency index is 2.60.

##### Phoneme blending

In the phoneme blending task, participants had to put together phoneme sounds to compose a non-word. The number of correctly blended phonemes (Phonemes_Acc_) and the total time in seconds for each non-word (Phonemes_Time_) were calculated and considered.

##### Rapid automatized naming

Rapid automatized naming (RAN) test of letters (RAN_Letters_) and colors (RAN_Colors_) were administered. In RAN_Letters_ and in RAN_Colors_, participants had to name letters and colors aloud as quickly and accurately as possible, respectively. Total time in seconds was considered for each task.

### Treatment

Direct current was delivered by a battery driven, direct current stimulator (BrainStim stimulation by E.M.S. s.r.l.—Bologna, Italy) *via* a pair of identical, rectangular (35 cm^2^) saline-soaked sponge electrodes held fastened by elastic bands. According to the International 10–20 System, the anodal electrode was positioned on the site corresponding to PO7, situated over the left parieto-occipital areas, specifically between left occipito-temporal and left temporo-parietal regions. Conversely, the cathodal electrode was placed on the right side of the parieto-occipital areas, corresponding to PO8, situated between right occipito-temporal and right temporo-parietal regions.

In line with previous studies on reading (Costanzo et al., 2016b,^2019^; Lazzaro et al., 2021c), we applied the left anodal/right cathodal tDCS montage. This methodological choice was mainly based on two reasons: (i) the well-known under activation of a distributed left hemisphere brain network in children and adolescents with DD (for a review, see Richlan, 2020); (ii) the polarity-specific effects of tDCS on reading (Turker and Hartwigsen, 2022), documented by studies showing that only left anodal/right cathodal placement induces positive changes (Costanzo et al., 2016a; Lazzaro et al., 2021a). Indeed, since anode generally facilitates neuronal activity and the cathode usually inhibits it, this montage is expected to push processing toward the left hemisphere, enhancing left lateralization. As already stated in Lazzaro et al. (2021b), the electrodes were placed according to studies reporting the involvement of the parieto-occipital regions in whole-word representations (Graves et al., 2008), in reading morphologically complex words (Zweig and Pylkkänen, 2009) and during the comprehension of complex linguistic units (Jobard et al., 2007).

In the active tDCS condition, the current slowly increased during the first 30 s to 1 mA (ramp-up) and, at the end of the stimulation, the current slowly decreased to 0 mA during the last 30 s (ramp-down). Between the ramp-up and ramp-down, a constant current was delivered for 20 min, with a density of 0.04 mA/cm^2^.

In the sham tDCS condition, the same montage used in the active tDCS condition was applied, respectively left anodal PO7 and right cathodal PO8. The stimulation intensity was set at 1 mA, but the current was applied for 30 s and was ramped down without the participants’ awareness. For more details, see Lazzaro et al. (2021b).

### Statistical analyses

To evaluate a possible order effect of active tDCS and sham conditions, analyses of covariance (ANCOVAs) were run (see Supplementary Table 2 for details).

The data were first examined for assumptions of normality and homogeneity of variance.

According to the Shapiro–Wilk test, the distributions of reading speed raw scores (TEXT, HF, LF, and NW) were found to be non-Gaussian. The raw scores were log-transformed and normally distributed. Therefore, to evaluate the effect of treatments on reading speed, repeated measures analysis of covariance (RM ANCOVAs) were run on each reading measure with Condition (Active vs. Sham) and Time (T0, T1, T2, and T3) as within-subject factors, and Age as covariate. *Post hoc* analyses were performed using Fisher’s LSD test. Partial eta squares ($\eta_{p}^{2}$) were used as measures of effect sizes. Bonferroni’s correction [*p* 0.05/4 RM ANCOVAs = 0.0125] was applied for multiple comparisons.

Non-parametric analyses were applied to analyze reading accuracy raw scores (TEXT, HF, LF, and NW) because the measures were non-Gaussian even after log-transformation. Therefore, generalized estimating equations (GEE) – an extension of generalized linear models – were run. The reading accuracy of TEXT, HF, LF, and NW was analyzed by fitting repeated measures regressions, using Condition (Active vs. Sham) and Time (T0, T1, T2, and T3) as predictors, and Age as covariate.

Significant main effects or interactions were performed by GEE-based pairwise comparisons with the least-significant difference test correction for multiple comparisons (for the approach see Santarnecchi et al., 2013; Borghini et al., 2018). Bonferroni’s correction [*p* 0.05/4 GEE-based pairwise = 0.0125] was applied for multiple comparisons. Non-parametric analyses were also applied to analyze neuropsychological measures (see Supplementary Tables 3, 4 for details).

For post hoc comparisons, a *p*-value ≤ 0.05 was considered statistically significant.

For each reading speed measure, the difference between the score at baseline (T0) and the score at each time point (T1, T2, and T3), divided by the score at T0 and multiplied by 100 was considered [i.e., Changes at T1 (Δ_*T*1_): (T0–T1)/T0 × 100; Changes at T2 (Δ_*T*2_): (T0–T2)/T0 × 100; Changes at T3 (Δ_*T*3_): (T0–T3)/T0 × 100]. Whereas, for each reading accuracy measure, the difference between the score at each time point (T1, T2, and T3) and the score at baseline (T0), divided by the score at T0 and multiplied by 100 was considered [i.e., Changes at T1 (Δ_*T*1_): (T1–T0)/T0 × 100; Changes at T2 (Δ_*T*2_): (T2–T0)/T0 × 100; Changes at T3 (Δ_*T*3_): (T3–T0)/T0 × 100].

To evaluate a potential relation between age and changes between baseline and post-treatments (Δ_*T*1_, Δ_*T*2_, and Δ_*T*3_) in reading tasks (speed and/or accuracy), Spearman’s rank correlations (*rho*) were performed separately for active and sham tDCS condition on significant results identified by RM ANCOVAs and by GEE. Bonferroni’s correction was applied for multiple comparisons.

To evaluate a potential relation between neuropsychological measures at T0 (visual-spatial and verbal working-memory_Eff_, Phonemes_Acc_, Phonemes_Time_, RAN_Letters_, and RAN_Colors_) and changes between baseline and post-treatments (Δ_*T*1_, Δ_*T*2_, and Δ_*T*3_) in reading tasks (speed and/or accuracy), partial Spearman’s rank correlations (rho) were performed separately for active and sham tDCS condition, controlling for age, on significant results identified by RM ANCOVAs and by GEE. Bonferroni’s Correction was applied for multiple comparisons.

## Results

### Effects of treatment on reading speed

Table 2 depicts means (SDs) of the main effect of Condition, Time, and the interaction Condition × Time for TEXT, HF, LF, and NW measures for both speed and accuracy.

**TABLE 2:** Means (SDs) of the main effect of condition, time and of the condition × time interaction for TEXT, HF, LF, and NW measures for both accuracy and speed.

Reading tasks		Condition		Time				Condition × time							
								Active tDCS				Sham tDCS			
		Active tDCS	Sham tDCS	T0	T1	T2	T3	T0	T1	T2	T3	T0	T1	T2	T3
TEXT	Accuracy^a^	93.65 (5.83)	93.17 (6.62)	93.19 (6.30)	93.31 (5.96)	93.58 (6.36)	93.57 (6.45)	93.84 (6.80)	93.70 (5.03)	93.55 (6.25)	94.03 (5.44)	93.04 (5.91)	92.92 (6.85)	93.62 (6.61)	93.11 (7.42)
	Speed^b^	55.99 (29.83)	55.43 (30.03)	59.73 (31.84)	55.25 (30.93)	55.33 (29.11)	52.52 (27.86)	59.38 (31.71)	56.95 (31.23)	54.44 (28.01)	53.20 (29.76)	60.08 (32.65)	53.55 (31.21)	56.22 (30.73)	51.85 (26.46)
HF	Accuracy^a^	92.76 (9.19)	93.03 (8.61)	93.39 (8.62)	92.19 (10.56)	92.66 (9.46)	93.35 (6.63)	92.81 (10.46)	92.19 (9.73)	92.71 (10.05)	93.33 (6.54)	93.96 (6.47)	92.19 (11.55)	92.61 (9.04)	93.37 (6.86)
	Speed^c^	24.49 (14.42)	25.35 (15.01)	25.65 (15.27)	24.22 (13.88)	24.91 (15.30)	24.92 (14.67)	25.79 (15.29)	24.25 (13.66)	23.27 (14.21)	24.67 (15.27)	25.50 (15.58)	24.18 (14.38)	26.55 (16.46)	25.17 (14.37)
LF	Accuracy^a^	87.03 (13.00)	86.19 (14.25)	86.88 (11.48)	85.36 (14.92)	86.65 (14.82)	87.56 (13.28)	87.08 (10.60)	87.60 (11.74)	86.15 (15.16)	87.29 (14.74)	86.67 (12.53)	83.13 (17.51)	87.16 (14.79)	87.83 (11.96)
	Speed^c^	34.78 (18.75)	34.81 (19.73)	36.15 (20.30)	33.55 (18.61)	34.71 (19.54)	34.77 (18.81)	35.83 (19.76)	34.38 (18.22)	33.73 (18.45)	35.17 (19.67)	36.46 (21.25)	32.72 (19.25)	35.68 (20.93)	34.37 (18.31)
NW	Accuracy^a^	81.07 (16.65)	79.33 (19.30)	80.21 (17.16)	78.02 (19.05)	80.51 (18.81)	82.06 (17.24)	81.98 (16.58)	80.52 (16.00)	78.85 (18.98)	82.92 (15.62)	78.44 (17.89)	75.52 (21.73)	82.16 (18.89)	81.20 (19.02)
	Speed^c^	38.61 (15.28)	37.67 (14.77)	40.13 (16.20)	37.52 (14.21)	37.81 (14.58)	37.11 (15.22)	41.96 (17.87)	36.96* (12.64)	38.52 (15.20)	37.00* (15.41)	38.29 (14.48)	38.08 (15.87)	37.09 (14.22)	37.23 (15.35)

^a^Percentage of accuracy, calculated as accuracy/total number of words × 100. ^b^Seconds/syllables × 100. ^c^Seconds. HF, high-frequency words; LF, low-frequency words; NW, non-words; T0, baseline; T1, immediately post-treatment; T2, 1 week later; T3, 1 month later.

*Significant difference from T0, p < 0.01.

Covarying for age, RM ANCOVA results on NW reading speed showed that the Condition effect [*F*(1,22) = 1.01, *p* = 0.33, $\eta_{p}^{2}=0.04$] and the Time effect [*F*(3,66) = 1.17, *p* = 0.33, $\eta_{p}^{2}=0.05$] were not significant, while the Condition × Time interaction was significant after Bonferroni’s correction [*F*(3,66) = 4.09, *p* = 0.01, $\eta_{p}^{2}=0.16$]. *Post hoc* analyses demonstrated that following active tDCS, reading times decreased after the end of treatment (T0 vs. T1: *p* = 0.012), and 1 month after the end of the treatment (T0 vs. T3: *p* = 0.002) compared with baseline. However, following sham tDCS, no significant differences were observed immediately after (T0 vs. T1: *p* = 0.48), nor 1 week later the end of the treatment (T0 vs. T2: *p* = 0.34), nor 1 month later the end of the treatment (T0 vs. T3: *p* = 0.21) compared with baseline (see Table 2).

Covarying for age, no effects emerged for TEXT [Condition effect: *F*(1,22) = 1.47, *p* = 0.24, $\eta_{p}^{2}=0.06$; Time effect: *F*(3,66) = 2.28, *p* = 0.09, $\eta_{p}^{2}=0.09$; Condition × Time interaction: *F*(3,66) = 0.43, *p* = 0.73, $\eta_{p}^{2}=0.02$] nor for LF [Condition effect: *F*(1,22) = 0.59, *p* = 0.45, $\eta_{p}^{2}=0.03$; Time effect: *F*(3,66) = 1.66, *p* = 0.18, $\eta_{p}^{2}=0.07$; Condition × Time interaction: *F*(3,66) = 0.98, *p* = 0.41, $\eta_{p}^{2}=0.04$].

Similarly, Condition effect [*F*(1,22) = 0.05, *p* = 0.83, $\eta_{p}^{2}=0.04$] and Time effect [*F*(3,66) = 1.05, *p* = 0.38, $\eta_{p}^{2}=0.05$] were not significant in HF reading speed, while the Condition × Time interaction was found significant.

Similarly, the Condition effect [*F*(1,22) = 0.05, *p* = 0.83, $\eta_{p}^{2}=0.04$] and the Time effect [(3,66) = 1.05, *p* = 0.38, $\eta_{p}^{2}=0.05$] were not significant with respect to the reading speed of HF. In contrast, the Condition × Time interaction was found to be significant [*F*(3,66) = 2.94, *p* = 0.04, $\eta_{p}^{2}=0.12$]. *Post hoc* analysis showed no significant results when comparing the active and sham conditions at different time points [*p* always > 0.05].

### Effects of treatment on reading accuracy

Covarying for age (see Table 2), GEE model results showed no significant effects for TEXT [Condition effect: Wald χ^2^(1) = 0.02, *p* = 0.88; Time effect: Wald χ^2^(3) = 1.63, *p* = 0.65; Condition × Time interaction: Wald χ^2^(3) = 0.12, *p* = 0.98], HF [Condition effect: Wald χ^2^(1) = 0.02, *p* = 0.89; Time effect: Wald χ^2^(3) = 5.70, *p* = 0.13; Condition × Time interaction: Wald χ^2^(3) = 1.07, *p* = 0.79], LF [Condition effect: Wald χ^2^(1) = 0.92, *p* = 0.34; Time effect: Wald χ^2^(3) = 0.48, *p* = 0.92; Condition × Time interaction: Wald χ^2^(3) = 3.41, *p* = 0.33], nor NW [Condition effect: Wald χ^2^(1) = 2.62, *p* = 0.11; Time effect: Wald χ^2^(3) = 4.43, *p* = 0.22; Condition × Time interaction: Wald χ^2^(3) = 0.71, *p* = 0.87].

### Correlations between age and reading

In the active tDCS condition, significant and negative correlations were found between age and Δ_T1_ and Δ_T3_ NW reading speed (respectively, rho = −0.50, *p* = 0.012 and rho = −0.42, *p* = 0.041), whereby as age decreased, greater improvement in NW reading speed was observed. No correlation between age and Δ_T2_ NW reading speed emerged (rho = −0.38, *p* = 0.07). After Bonferroni’s correction (*p* 0.05/3Δ = 0.016), a negative correlation between age and Δ_T1_ NW reading speed survived.

In the sham tDCS condition, no correlations between age and Δ_T1_, Δ_T2_, Δ_T3_ NW reading speed emerged (respectively, rho = 0.04, *p* = 0.84; rho = 0.23, *p* = 0.29; rho = 0.40, *p* = 0.05).

See Supplementary material for the correlations between age and non-significant reading measures identified by RM ANCOVAs and by GEE (Supplementary Table 5).

### Correlations between neuropsychological measures and reading

In the active tDCS condition, significant negative correlations were found between verbal working-memory_Eff_ at T0 and Δ_T1_, Δ_T2_ and Δ_T3_ NW reading speed (respectively, *p* < 0.005, *p* < 0.002, and *p* < 0.006), so the lower the verbal working memory efficiency at T0 (more impaired), the greater the improvement.

Moreover, significant positive correlations were found between Phonemes_Time_ at T0 and Δ_T1_ and Δ_T3_ NW reading speed (respectively, *p* < 0.029 and *p* < 0.043), so the longer the time taken to merge the non-word at T0 (more impaired), the greater the improvement.

After Bonferroni’s correction [*p* 0.05/3Δ × 6 measures = 0.0028], a negative correlation between verbal working-memory_Eff_ at T0 and Δ_T2_ NW reading speed survived.

No further correlations emerged [*p* always > 0.05]. The other correlations between neuropsychological measures at T0 and Δ_T1_, Δ_T2_, Δ_T3_ NW reading speed in the active tDCS and sham tDCS condition are shown in Table 3.

**TABLE 3:** Correlations between neuropsychological measures at T0 and Δ_T1_, Δ_T2_, Δ_T3_ NW reading speed, controlling for age, in the active and sham tDCS conditions.

Neuropsychological measures at T0							
NW speed		Working-memory_Eff_		Phoneme blending		RAN	
		Visual-spatial	Verbal	Accuracy^a^	Time^b^	Letters^b^	Colors^b^
		*(Rho)*	*(Rho)*	*(Rho)*	*(Rho)*	*(Rho)*	*(Rho)*
Δ_T1_	Active tDCS	–0.22	−0.57**	–0.10	0.46*	0.10	–0.08
Δ_T2_		–0.02	−0.62^**∧^	–0.18	0.38	–0.05	–0.02
Δ_T3_		–0.15	−0.55**	–0.15	0.43*	–0.13	–0.40
Δ_T1_	Sham tDCS	0.19	–0.13	0.03	0.21	0.08	0.07
Δ_T2_		–0.06	–0.24	–0.17	0.14	0.16	0.33
Δ_T3_		0.36	0.20	–0.07	–0.02	–0.20	–0.21

^a^Number of phonemes. ^b^Seconds. RAN, rapid automatized naming; NW, non-words; Δ_T1_, changes at T1; Δ_T2_, changes at T2; Δ_T3_, changes at T3.

*p ≤ 0.05; **p ≤ 0.01; ^∧^significant after Bonferroni’s correction (p ≤ 0.0028).

See Supplementary material for the correlations between neuropsychological measures at T0 and non-significant reading measures identified by RM ANCOVAs and by GEE (Supplementary Tables 6, 7).

Moreover, the relation between neuropsychological measures at T0 and NW reading speed at T0 has been explored (see Supplementary Table 8).

## Discussion

To date, this is the first RCT study of 24 children and adolescents with DD to test the effectiveness of multiple consecutive daily sessions of tDCS through a crossover design.

We found that only five consecutive daily sessions of active left anodal/right cathodal tDCS over parieto-occipital regions significantly improved NW reading speed at post-treatment follow-ups compared with baseline. Our previous studies (Costanzo et al., 2016b,^2019^) demonstrated that three sessions per week for 6 weeks (for a total of 18 sessions) of left anodal/right cathodal tDCS combined with reading training improved NW reading speed by an average of 15 s compared to baseline. Compared with these previous studies (Costanzo et al., 2016b,^2019^), in the present study we found that the average improvement in speed in NW reading compared with baseline is 5 s, which is 3 times lower than that previously obtained after 18 sessions of tDCS combined with reading training. As discussed (Lazzaro et al., 2021b), possible explanations for the less robust effect of non-invasive brain stimulation in the present study could be related to the reduced number of tDCS sessions compared with the previous studies and the absence of reading training associated with tDCS. The effect found is consistent with studies showing that the results of non-invasive brain stimulation depend not only on current intensity but also on the duration of stimulation (Nitsche and Paulus, 2000).

In addition, the present study extended to 1 month the positive effect of active tDCS on NW reading speed previously found at 1 week after the end of treatment (Lazzaro et al., 2021b). It should be noted that 5 sessions were sufficient to maintain up to 1 month the effect found immediately at the end of the treatment, similar to what happened after 18 sessions of tDCS combined with cognitive training (Costanzo et al., 2016b,^2019^).

By analyzing the two results together, we provided evidence that a treatment of a few sessions, without training, has a stable effect, which is maintained at 1 month, although weaker.

Regarding correlations, we found that as age decreased, the NW reading speed improved immediately after and 1 month after the end of the active tDCS condition. A large body of literature has shown that age – and the related thickness of the skull, maturation of brain regions, hormonal disturbances, and neurotransmitter activity – is a determinant of neuroplasticity (Vergallito et al., 2022). Neural plasticity is one of the main mechanisms involved in the stimulation effects, which depends on the personal propensity to induce plasticity (Bandeira et al., 2021). This propensity tends to be more significant at a young age and decreases throughout life with a lower tendency to occur in later life (Ridding and Ziemann, 2010; Freitas et al., 2013).

However, the present results differ from those of our previous study (Lazzaro et al., 2021c), in which we documented that older children in the active tDCS group improved word reading speed more than younger children in each follow-up. One possible explanation for this discrepancy can be found in the methodological differences between the studies. In fact, in the previous study (Lazzaro et al., 2021c), tDCS was administered together with reading training, so it can be hypothesized that older children were able to use more complex cognitive strategies, taking more advantage of the cognitive training associated with tDCS. Therefore, the effects of tDCS would have been eventually triggered and critically reinforced by ongoing cognitive strategies, probably more exploited by older children, accelerating progress during training.

Considering the correlations between neuropsychological measures at baseline and reading improvement, we found that when verbal working memory and phoneme blending were worse at baseline, NW reading speed improved more immediately after the active tDCS condition and at long-term. Together with the verbal working memory, phonological skills are one of the main predictors of reading development (Melby-Lervåg et al., 2012), especially non-word reading, for which grapheme-to-phoneme mapping is required. It can be hypothesized that the children who have greater difficulty in phonological measures, such as phoneme blending and verbal working memory, are also the one who have greater difficulty in NW reading at baseline. Therefore, those who had greater impairment in phonological skills and verbal working memory, which mirror reading skills, were more likely to have increased reading abilities after active tDCS than those who had a reading deficit but lesser severity.

Taking together our results on correlations, we showed that the improvement in NW reading speed after active tDCS, which is the most consistent finding in our studies (Costanzo et al., 2016b,^2019^; Lazzaro et al., 2021b), is associated with age, level of phonological skills, and verbal working memory achieved by participants at baseline.

Our study had some limitations.

The first limitation was the absence of a direct comparison between the current tDCS protocol and longer stimulation protocols in which multiple tDCS sessions are offered without reading training.

Similarly, a direct comparison of tDCS protocols with and without reading training would be needed to clarify the magnitude of the effect of tDCS when the neural population is preactivated by training at the time of its application compared with when brain stimulation is administered alone. Further, although there is agreement on the usefulness of increasing cortical excitability in left hemispheric regions involved in reading processes, further studies investigating the effects of stimulation in contralateral areas are needed.

Another limitation was the lack of non-verbal neuropsychological measures (such as attention and visual-spatial perception), as we mainly focused on verbal neuropsychological measures and their relation to reading to understand how reading can be modulated by tDCS.

In addition, in the context of the promises of tDCS interventions, the role of participants’ self-agency should be considered in further studies. Indeed, proposing stand-alone tDCS-based treatments could have implications for beliefs and self-representations. If improvement is achieved through external stimulation, without the active involvement of participant playing a passive role, there is a risk that the participant will lose self-confidence as an agent who is responsible for the results achieved and able to manage cognitive resources. The present study, despite the considerations just made, aimed to precisely measure the specific influence of tDCS in improving reading skills in the absence of additional stimuli, thus not involving paired task. Future studies, however, should consider the role of participants in the tDCS interventions.

Moreover, because DD can evolve over time with different clinical manifestations, a limitation of this study may be the consideration of a wide age range by including children and adolescents. However, this limitation was partially overcome by considering participants’ age as a confounding variable and including it as a covariate in all models.

## Conclusion

In conclusion, the current crossover RCT contributed to (i) enforce previous effects of tDCS, including long-term effects, on NW reading speed; (ii) understand the effect of age on tDCS delivered without concomitant training; and (iii) consider neuropsychological processes at baseline as one of the relevant factors contributing to reading improvement after tDCS.

Although we are far from identifying the most effective tDCS-based protocol, our results may have high translational power if we consider that our short and intensive intervention turns out to have beneficial consequences even in the long-term.

In fact, an elective first-choice treatment for children and adolescents with DD has not yet been demonstrated. Programs usually delivered involve at least 6 months of weekly meetings, with a high dropout rate, unsustainable costs to parents or the health care system, and long-term effects that are not well verified.

With these premises, sustainable and cost-effective interventions for DD are urgently needed. Considering our results, tDCS may indeed represent a neurobiologically based, low-cost, and easy-to-implement therapeutic option with long-term effects for children and adolescents with DD.

## Data availability statement

The raw data supporting the conclusions of this article will be made available by the authors, without undue reservation.

## Ethics statement

The studies involving human participants were reviewed and approved by the Bambino Gesù Children’s Hospital, IRCCS. Written informed consent to participate in this study was provided by the participants’ legal guardian/next of kin.

## Author contributions

FC, SV, and DM designed the study. FC, GL, CV, and SR collected the data. AB, GL, and DM worked on data analyses. AB and GL drafted the manuscript, with support of DM and SV. DM and SV supervised the study. All authors contributed to the article and approved the submitted version.
