# Supplementary material for: Effects of a short and intensive transcranial direct current stimulation treatment in children and adolescents with developmental dyslexia: A crossover clinical trial
Source: Front Psychol. 2022 Sep 9;13:986242. doi: 10.3389/fpsyg.2022.986242 (PMC9500580; doi:10.3389/fpsyg.2022.986242)
Supplement: Supplementary file 1 [file Table_1.docx]

**Supplementary Materials**

# Reading tasks

Eight different versions of four sets of stimuli (text, 2 lists of words and 1 list of non-word) were submitted to 20 typical readers (9 females; M = 11.23 yrs, SD = 2.14 yrs) in a behavioural pre-test. Each typical reader had to read aloud as rapid and accurate as possible the following reading tasks:

-8 texts of over 400 syllables long (TEXT);

-8 lists of 20 high frequency words (HF - 10 trisyllabic and 10 bisyllabic);

-8 lists of 20 low frequency words (LF - 10 trisyllabic and 10 bisyllabic);

-8 lists of 20 non-word (NW - 10 trisyllabic and 10 bisyllabic) created by rearranging the character string of real word items.

TEXT was written with Time New Roman font, size 13, single-spaced, on a white sheet of A4 paper. HF, LF, NW were arranged in 20-items columns, written with Times New Roman font, size 13, single-spaced, on a white sheet of A4 paper. TEXT derived from an Italian novel (Calvino, 1963). Items in HF list and LF list were matched for Italian written word frequency, number of letters and syllables, bigram frequency (according to CoLFIS, http://www. istc.cnr.it/material/database/colfis) and mean onset reaction time (Barca et al., 2002).

Considering speed, the total time (in terms of seconds) taken to read HF, LF and NW was measured. For TEXT, reading speed was calculated by dividing the total time (in terms of seconds) for reading completion by the total number of syllables spoken and multiplied by 100.

Considering accuracy, an error point was assigned in the presence of substitution, omission, addition of letters and in case of self-correction or hesitation during reading. For all reading tasks (TEXT, HF, LF, NW), the percentage of accuracy was considered, calculated by multiplying the ratio of the number of correctly read stimuli to the total number of stimuli presented by 100.

The accuracy and reading speed of each set of stimuli were compared, and the following equivalent stimuli were selected (see Table S1 for the means (SDs)):

- out of 8 versions, 5 texts [Accuracy: F(4, 76) = 1.63, *p* = 0.17, η_p_^2^ = 0.08; Speed: F(4, 76) = 1.56, *p* = 0.19, η_p_^2^ = 0.08];

- out of 8 versions, 5 lists of HF [Accuracy: F(4, 76) = 2.32, *p* = 0.06, η_p_^2^ = 0.11; Speed: F(4, 76) = 0.34, *p* = 0.85, η_p_^2^ = 0.02];

- out of 8 versions, 5 lists of LF [Accuracy: F(4, 76) = 1.07, *p* = 0.38, η_p_^2^ = 0.05; Speed: F(4, 76) = 1.05, *p* = 0.39, η_p_^2^ = 0.05];

- out of 8 versions, 7 lists of NW [Accuracy: F(6, 114) = 1.23, *p* = 0.29, η_p_^2^ = 0.06; Speed: F(6, 114) = 0.39, *p* = 0.88, η_p_^2^ = 0.02].

---Table S1---

# Preliminary analyses

To evaluate possible order effect, Analyses of Covariance (ANCOVAs) were performed on each reading and neuropsychological measure at baseline (T0) with Condition (Active vs Sham) as within-subject factor, Group (A_S_Group_ vs S_A_Group_) as between-subject factor, and Age as covariate. Post-hoc analyses were performed by using Fisher’s LSD test. Partial eta squares (η_p_^2^) were used as measures of effect sizes.

Non-parametric analyses were applied to analyse neuropsychological measures (visual-spatial and verbal working-memory_Eff_, Phonemes_Acc,_ Phonemes_Time_, RAN_Letters_ and RAN_Colours_) because the measures were non-Gaussian even after log-transformation. Therefore, Generalized Estimating Equations (GEE) – an extension of generalized linear models – were run. Raw scores of neuropsychological measures were analysed by fitting repeated-measures regressions, using Condition (Active vs Sham) and Time (T0, T1, T2, T3) as predictors, and Age as covariate. Significant main effects or interactions were performed by GEE-based pairwise comparisons with the least-significant difference test correction for multiple comparisons (for a similar approach see Santarnecchi et al., 2013; Borghini et al., 2018). Bonferroni’s Correction [*p* 0.05/6 GEE-based pairwise = 0.008] was applied for multiple comparisons.

A *p* value ≤ 0.05 was considered statistically significant.

To evaluate a potential relation between neuropsychological measures at T0 (visual-spatial and verbal working-memory_Eff_, Phonemes_Acc,_ Phonemes_Time_, RAN_Letters_ and RAN_Colours_) and percentages of change in TEXT, HF, LF, and NW reading measures (speed and accuracy), Partial Spearman's rank correlations (*rho*) were performed separately for active and sham tDCS condition, controlling for age.

For all correlations, a p value ≤ 0.0028 was considered (after Bonferroni’s Correction, *p* = 0.05/18 = 0.0028).

To evaluate a potential relation between neuropsychological measures at T0 (visual-spatial and verbal working-memory_Eff_, Phonemes_Acc,_ Phonemes_Time_, RAN_Letters_ and RAN_Colours_) and NW reading speed at T0, Partial Spearman's rank correlations (*rho*) were performed separately for active and sham tDCS condition, controlling for age.

For all correlations, a p value ≤ 0.0083 was considered (after Bonferroni’s Correction, 0.05/6 = 0.0028).

# Results

## Order effect on reading speed

Table S2 depicts means (SDs) of the main effect of Group and the Condition x Group interaction for TEXT, HF, LF and NW measures for both speed and accuracy at T0.

Covarying for age, results on ANCOVA showed that no effects emerged for TEXT [Group effect: F(1, 21) = 0.44, *p* = 0.51, η_p_^2^ = 0.02, Condition x Group interaction: F(1, 21) = 1.67, *p* = 0.21, η_p_^2^ = 0.07], LF [Group effect: F(1, 21) = 0.14, *p* = 0.71, η_p_^2^ = 0.01; Condition x Group interaction: F(1, 21) = 0.29, *p* = 0.60, η_p_^2^ = 0.01], and for NW [Group effect: F(1, 21) = 0.72, *p* = 0.41, η_p_^2^ =0.03; Condition x Group interaction: F(1, 21) = 1.34, *p* = 0.26, η_p_^2^ = 0.06]. In HF, the Group effect was not significant [F(1, 21) = 0.07, *p* = 0.80, η_p_^2^ = 0.0003], while the Condition x Group interaction was found significant [F(1, 21) = 5.88, *p* = 0.02, η_p_^2^ = 0.22]. Post-hoc analysis showed a significant difference between groups for active tDCS conditions at T0 [*p* = 0.025].

- 1. **Order effect on reading accuracy**

Covarying for age, results on ANCOVA showed that no effects emerged for TEXT [Group effect: F(1, 21) = 0.48, *p* = 0.50, η_p_^2^ = 0.02; Condition x Group interaction: F(1, 21) = 1.46, *p* = 0.24, η_p_^2^ = 0.06], HF [Group effect: F(1, 21) = 0.30, *p* = 0.59, η_p_^2^ = 0.14; Condition x Group interaction: F(1, 21) = 1.32, *p* = 0.26, η_p_^2^ = 0.06], LF [Group effect: F(1, 21) = 1.24, *p* = 0.28, η_p_^2^ = 0.03; Condition x Group interaction: F(1, 21) = 0.04, *p* = 0.85, η_p_^2^ = 0.002], and NW [Group effect: F(1, 21) = 0.43, *p* = 0.52, η_p_^2^ = 0.02; Condition x Group interaction: F(1, 21) = 0.61, *p* = 0.44, η_p_^2^ = 0.03]. See Table S2.

---Table S2---

- 1. **Order effect on neuropsychological measures**

Table S3 depicts means (SDs) of the main effect of Group and the interaction Condition x Group for neuropsychological measures.

Covarying for age, results on ANCOVA showed that no effects emerged for visual-spatial working-memory_Eff_ [Group effect: F(1, 21) = 0.45, *p* = 0.83, η_p_^2^ = 0.002; Condition x Group interaction: F(1, 21) = 4.12, *p* = 0.06, η_p_^2^ = 0.16], Phonemes_Acc_ [Group effect: F(1, 21) = 0.10, *p* = 0.76, η_p_^2^ = 0.004; Condition x Group interaction: F(1, 21) = 3.14, *p* = 0.09, η_p_^2^ = 0.13] and RAN_Colours_ [Group effect: F(1, 21) = 0.74, *p* = 0.40, η_p_^2^ = 0.03; Condition x Group interaction: F(1, 21) = 0.01, *p* = 0.92, η_p_^2^ = 0.0005].

In verbal working-memory_Eff_, the Group effect was not significant [F(1, 21) = 2.74, *p* = 0.11, η_p_^2^ = 0.12], while the Condition x Group interaction was found significant [F(1, 21) = 13.49, *p* = 0.001, η_p_^2^ = 0.39]. Post-hoc analysis showed a significant difference between groups in the active tDCS conditions at T0 [*p* = 0.0001]. Moreover, a significant difference between active tDCS and sham tDCS conditions emerged only in the A_S_Group_ [*p* = 0.004].

In Phonemes_Time_, the Group effect was not significant [F(1, 21) = 1.20, *p* = 0.29, η_p_^2^ = 0.05], while the Condition x Group interaction was found significant [F(1, 21) = 18.16, *p* = 0.0003, η_p_^2^ = 0.46]. Post-hoc analysis showed a significant difference between active tDCS and sham tDCS conditions in the A_S_Group_ [*p* = 0.003] and in the S_A_Group_ [*p* = 0.01].

In RAN_Letters_, the Group effect was not significant [F(1, 21) = 0.82, *p* = 0.37 η_p_^2^ = 0.04], while the Condition x Group interaction was found significant [F(1, 21) = 4.54, *p* = 0.05, η_p_^2^ = 0.18]. Post-hoc analysis showed a significant difference between groups in the active tDCS conditions at T0 [*p* = 0.013].

---Table S3---

## Effects of treatment on neuropsychological measures

Covarying for age, results on GEE model in verbal working-memory_Eff_ showed that the Condition effect [Wald χ^2^(1) = 0.51, *p* = 0.47] and the Condition x Time interaction [Wald χ^2^(3) = 1.65, *p* = 0.65] were not significant, while the Time effect was found significant [Wald χ^2^(3) =10.09, *p* = 0.02]. However, post-hoc analysis did not show significant results when comparing baseline with each time point [T0 v*s* T1: mean difference = – 0.22, *p* = 0.87; T0 v*s* T2: mean difference = – 0.20, *p* = 0.94; T0 v*s* T3: mean difference = – 0.26, *p* = 0.85].

As well, covarying for age, results on GEE model in Phonemes_Time_ showed that the Condition effect [Wald χ^2^(1) = 0.14, *p* = 0.71] and the Condition x Time interaction [Wald χ^2^(3) = 4.61, *p* = 0.20] were not significant, while the Time effect was found significant [Wald χ^2^(3) = 17.10, *p* = 0.001]. However, post-hoc analysis did not show significant results when comparing baseline with each time point [T0 v*s* T1: mean difference = 3.17, *p* = 0.94; T0 v*s* T2: mean difference = 6.04, *p* = 0.90; T0 v*s* T3: mean difference = 3.92, *p* = 0.91].

Covarying for age, results on GEE model showed no significant effects for visual-spatial working-memory_Eff_ [Condition effect: Wald χ^2^(1) = 1.71, *p* = 0.19; Time effect: Wald χ^2^(3) = 1.31, *p* = 0.73; Condition x Time interaction: Wald χ^2^(3) = 6.27, *p* = 0.10], Phonemes_Acc_ [Condition effect: Wald χ^2^(1) = 0.28, *p* = 0.60; Time effect: Wald χ^2^(3) = 5.87, *p* = 0.12; Condition x Time interaction: Wald χ^2^(3) = 6.20, *p* = 0.10], RAN_Letters_ [Condition effect: Wald χ^2^(1) = 0.68, *p* = 0.41; Time effect: Wald χ^2^(3) = 6.54, *p* = 0.09; Condition x Time interaction: Wald χ^2^(3) = 2.99, *p* = 0.39], and RAN_Colours_ [Condition effect: Wald χ^2^(1) = 0.47, *p* = 0.49; Time effect: Wald χ^2^(3) = 1.52, *p* = 0.68; Condition x Time interaction: Wald χ^2^(3) = 2.08, *p* = 0.55]. See Table S4.

---Table S4---

- 1. **Supplementary correlations**

Table S5 shows correlations between age and ∆T1, ∆T2, ∆T3 for TEXT, HF, LF, and NW reading accuracy and speed in the active and sham tDCS conditions.

---Table S5---

Table S6 shows correlations between neuropsychological measures at T0 and ∆T1, ∆T2, ∆T3 for TEXT, HF, LF, and NW reading accuracy and speed, controlling for age, in the active tDCS condition.

Table S7 shows correlations between neuropsychological measures at T0 and ∆_T1_, ∆_T2_, ∆_T3_ for TEXT, HF, LF, and NW reading accuracy and speed, controlling for age, in the sham tDCS condition.

---Table S6---

---Table S7---

Table S8 shows correlations between neuropsychological measures at T0 and NW reading speed at T0, controlling for age, in the active and sham tDCS conditions.

---Table S8---

Table S1. Means (SDs) of reading accuracy and speed for each version of the selected set of stimuli (TEXT, HF, LF, and NW).

| Reading Tasks | | #1 | #2 | #3 | #4 | #5 | #6 | #7 |
| --- | --- | --- | --- | --- | --- | --- | --- | --- |
| TEXT | Accuracy^a^ | 97.78  (1.56) | 97.68  (1.52) | 97.61  (2.10) | 98.09  (1.64) | 98.07  (1.65) | - | - |
|  | Speed^b^ | 26.82  (10.68) | 28.79  (11.67) | 28.30  (11.92) | 27.40  (11.19) | 28.46  (11.16) | - | - |
| HF | Accuracy^a^ | 98.00  (2.64) | 96.00  (5.09) | 97.63  (3.49) | 97.13  (4.39) | 96.13  (3.67) | - | - |
|  | Speed^c^ | 12.00  (4.97) | 12.50  (4.86) | 12.20  (5.86) | 12.10  (4.76) | 12.65  (4.61) | - | - |
| LF | Accuracy^a^ | 93.13  (6.00) | 95.25  (4.28) | 93.88  (6.15) | 94.13  (5.47) | 93.13  (6.22) | - | - |
|  | Speed^c^ | 15.85  (7.35) | 15.50  (7.39) | 16.90  (8.11) | 16.50  (7.60) | 15.85  (7.87) | - | - |
| NW | Accuracy^a^ | 93.50  (6.56) | 91.00  (7.80) | 89.50  (11.37) | 89.88  (10.59) | 89.25  (9.63) | 90.38  (11.04) | 92.38  (6.61) |
|  | Speed^c^ | 20.75  (8.73) | 21.75  (8.74) | 22.25  (11.64) | 21.50  (10.46) | 21.50  (8.95) | 22.05  (10.05) | 21.25  (10.62) |
| ^a^ Percentage of accuracy, calculated as accuracy/total number of words x 100; ^b^ Seconds/syllables x 100; ^c^ Seconds. HF, High-Frequency words; LF, Low-Frequency words; NW, Non-words. | | | | | | | | |

Table S2. Means (SDs) of the main effect of Group and the Condition x Group interaction for TEXT, HF, LF and NW measures for both speed and accuracy at T0.

|  | |  | Group | |  | Condition x Group | | | | |
| --- | --- | --- | --- | --- | --- | --- | --- | --- | --- | --- |
| Reading Tasks | |  | A_S_Group_ | S_A_Group_ |  | A_S_Group_ | |  | S_A_Group_ | |
|  |  |  |  |  |  | Active tDCS | Sham tDCS |  | Active tDCS | Sham tDCS |
| TEXT | Accuracy^a^ |  | 92.70  (4.17) | 93.68  (8.11) |  | 93.28  (3.89) | 92.12  (4.45) |  | 93.39  (9.06) | 93.96  (7.16) |
|  | Speed^b^ |  | 67.69  (37.70) | 48.77  (21.59) |  | 72.28  (37.28) | 63.09  (38.12) |  | 46.47  (18.70) | 51.07  (24.48) |
| HF | Accuracy^a^ |  | 92.71  (6.74) | 94.07  (9.89) |  | 92.71  (6.78) | 92.71  (6.70) |  | 92.92  (13.52) | 95.21  (6.26) |
|  | Speed^c^ |  | 29.71  (17.93) | 21.58  (11.55) |  | 31.50  (17.61) | 27.92  (18.24) |  | 20.08  (10.35) | 23.08  (12.74) |
| LF | Accuracy^a^ |  | 85.73  (9.52) | 88.02  (13.61) |  | 86.67  (9.00) | 84.79  (10.03) |  | 87.50  (12.39) | 88.54  (14.83) |
|  | Speed^c^ |  | 41.55  (22.05) | 30.75  (18.14) |  | 41.17  (21.36) | 41.92  (22.74) |  | 30.50  (17.26 | 31  (19.02) |
| NW | Accuracy^a^ |  | 77.51  (14.25) | 82.92  (19.81) |  | 79.38  (16.24) | 75.63  (12.25) |  | 84.58  (17.22) | 81.25  (22.40) |
|  | Speed^c^ |  | 45  (15.64) | 35.25  (15.68) |  | 48.50  (15.98) | 41.50  (15.29) |  | 35.42  (17.86) | 35.08  (13.50) |
| ^a^ Percentage of accuracy, calculated as accuracy/total number of words x 100; ^b^ Seconds/syllables x 100; ^c^ Seconds. HF, High-Frequency words; LF, Low-Frequency words; NW, Non-words; T0, baseline. | | | | | | | | | | |

Table S3. Means (SDs) of the main effect of Group and the Condition x Group interaction for neuropsychological measures at T0.

|  | |  | Group | |  | Condition x Group | | | | |
| --- | --- | --- | --- | --- | --- | --- | --- | --- | --- | --- |
| Neuropsychological  Tasks | |  | A_S_Group_ | S_A_Group_ |  | A_S_Group_ | |  | S_A_Group_ | |
|  |  |  |  |  |  | Active tDCS | Sham tDCS |  | Active tDCS | Sham tDCS |
| Working memory_Eff_ | Visual-spatial |  | 2.33  (0.64) | 2.50  (0.59) |  | 2.09  (0.70) | 2.57  (0.57) |  | 2.58  (0.48) | 2.43  (0.70) |
|  | Verbal |  | 2.21  (0.49) | 2.66  (0.63) |  | 1.93  (0.48) | 2.50^  (0.49) |  | 2.75**  (0.61) | 2.57  (0.64) |
| Phoneme Blending | Accuracy^a^ |  | 45.75  (14.25) | 53.29  (15.04) |  | 43.83  (15.34) | 47.67  (13.16) |  | 54.67  (14.03) | 51.92  (16.04) |
|  | Time^b^ |  | 20.21  (9.76) | 21.62  (20.62) |  | 26.03  (12.90) | 14.39^  (6.62) |  | 16.94  (14.96) | 26.31^  (26.28) |
| RAN | Letters^b^ |  | 3.68  (0.96) | 3.04  (0.95) |  | 3.84  (1.10) | 3.52  (0.81) |  | 2.92*  (0.85) | 3.17  (1.05) |
|  | Colours^b^ |  | 4.93  (1.67) | 4.01  (1.21) |  | 5.11  (1.42) | 4.75  (1.92) |  | 4.11  (1.32) | 3.90  (1.09) |
| ^a^ Number of Phonemes; ^b^ Seconds; RAN, Rapid Automatized Naming; T0, baseline; T1, immediately post-treatment; T2, 1-week later; T3, 1-month later.  * p < 0.05, ** p < 0.001; significantly different from active tDCS condition in the A_S_Group_;  ^ p < 0.01; significantly different from active tDCS condition. | | | | | | | | | | |

Table S4. Means (SDs) of the main effect of Condition, Time and of the Condition x Time interaction for each neuropsychological measure.

|  | | Condition | | Time | | | | Condition x Time | | | | | | | |
| --- | --- | --- | --- | --- | --- | --- | --- | --- | --- | --- | --- | --- | --- | --- | --- |
| Neuropsychological Tasks | |  |  |  | | | | Active tDCS | | | | Sham tDCS | | | |
|  |  | Active  tDCS | Sham  tDCS | T0 | T1 | T2 | T3 | T0 | T1 | T2 | T3 | T0 | T1 | T2 | T3 |
| Working memory_Eff_ | Visual-spatial | 2.60  (0.74) | 2.66  (0.67) | 2.42  (0.64) | 2.52  (0.50) | 2.83  (1.04) | 2.75  (0.66) | 2.33  (0.64) | 2.55  (0.45) | 2.77  (1.22) | 2.76  (0.66) | 2.50  (0.63) | 2.50  (0.54) | 2.90  (0.85) | 2.75  (0.66) |
|  | Verbal | 2.58  (0.56) | 2.66  (0.63) | 2.44  (0.62) | 2.69  (0.51) | 2.62  (0.70) | 2.74  (0.55) | 2.34  (0.68) | 2.69  (0.51) | 2.65  (0.45) | 2.64  (0.59) | 2.53  (0.55) | 2.68  (0.51) | 2.58  (0.95) | 2.84  (0.51) |
| Phoneme Blending | Accuracy^a^ | 51.15  (13.94) | 52.44  (12.82) | 49.52  (14.96) | 53.53  (14.06) | 52.62  (12.07) | 51.50  (12.44) | 49.25  (15.40) | 52.06  (15.77) | 51.38  (11.80) | 51.92  (12.77) | 49.79  (14.52) | 55  (12.34) | 53.86  (12.33) | 51.09  (12.10) |
|  | Time^b^ | 16.70  (9.87) | 18.56  (14.49) | 20.92  (17.07) | 18.03  (10.57) | 14.90  (7.45) | 16.68  (13.63) | 21.48  (14.43) | 17.25  (10.16) | 13.59  (6.57) | 14.47  (8.32) | 20.5  (19.70) | 18.80  (10.98) | 16.21  (8.33) | 18.88  (18.94) |
| RAN | Letters^b^ | 3.33  (1.05) | 3.35  (0.95) | 3.36  (1.00) | 3.49  (0.99) | 3.30  (1.00) | 3.21  (1.01) | 3.38  (1.07) | 3.39  (1.08) | 3.29  (1.02) | 3.27  (1.02) | 3.34  (0.93) | 3.58  (0.89) | 3.32  (0.98) | 3.16  (1.00) |
|  | Colours^b^ | 4.46  (1.49) | 4.40  (1.33) | 4.47  (1.51) | 4.50  (1.26) | 4.59  (1.68) | 4.16  (1.19) | 4.61  (1.43) | 4.16  (1.09) | 4.81  (2.22) | 4.27  (1.20) | 4.33  (1.59) | 4.84  (1.43) | 4.36  (1.13) | 4.06  (1.17) |
| ^a^ Number of Phonemes; ^b^ Seconds; RAN, Rapid Automatized Naming; T0, baseline; T1, immediately post-treatment; T2, 1-week later; T3, 1-month later. | | | | | | | | | | | | | | | |

Table S5. Correlations between age and ∆_T1_, ∆_T2_, ∆_T3_ for TEXT, HF, LF, and NW reading accuracy and speed in the active tDCS condition.

|  |  |  | Age | |
| --- | --- | --- | --- | --- |
|  | Reading Tasks |  | Active tDCS | Sham tDCS |
|  |  |  | *(Rho)* | *(Rho)* |
| TEXT | Accuracy^a^ | ∆_T1_ | 0.01 | 0.10 |
|  |  | ∆_T2_ | 0.14 | 0.22 |
|  |  | ∆_T3_ | 0.20 | 0.19 |
|  | Speed^b^ | ∆_T1_ | 0.14 | 0.09 |
|  |  | ∆_T2_ | -0.15 | 0.17 |
|  |  | ∆_T3_ | -0.31 | -0.02 |
| HF | Accuracy^a^ | ∆_T1_ | -0.17 | 0.06 |
|  |  | ∆_T2_ | -0.08 | 0.25 |
|  |  | ∆_T3_ | 0.07 | -0.03 |
|  | Speed^c^ | ∆_T1_ | 0.17 | 0.06 |
|  |  | ∆_T2_ | -0.13 | 0.35 |
|  |  | ∆_T3_ | 0.16 | 0.35 |
| LF | Accuracy^a^ | ∆_T1_ | 0.05 | -0.04 |
|  |  | ∆_T2_ | -0.03 | -0.09 |
|  |  | ∆_T3_ | 0.02 | -0.11 |
|  | Speed^c^ | ∆_T1_ | -0.34 | -0.65* |
|  |  | ∆_T2_ | -0.28 | 0.22 |
|  |  | ∆_T3_ | 0.09 | 0.27 |
| NW | Accuracy^a^ | ∆_T1_ | -0.20 | -0.09 |
|  |  | ∆_T2_ | -0.05 | 0.01 |
|  |  | ∆_T3_ | -0.17 | -0.01 |
| ^a^ Percentage of accuracy, calculated as accuracy/total number of words x 100; ^b^ Seconds/syllables x 100; ^c^ Seconds. HF, High-Frequency words; LF, Low-Frequency words; NW, Non-words; ∆_T1_, Changes at T1; ∆_T2_, Changes at T2; ∆_T3_, Changes at T3.  * p < 0.001 | | | | |

Table S6. Correlations between neuropsychological measures at T0 and ∆_T1_, ∆_T2_, ∆_T3_ for TEXT, HF, LF, and NW reading accuracy and speed, controlling for age, in the active tDCS condition.

|  |  |  | Active tDCS  Neuropsychological Tasks at T0 | | | | | | | | |
| --- | --- | --- | --- | --- | --- | --- | --- | --- | --- | --- | --- |
|  |  |  | Working memory_Eff_ | |  | Phoneme Blending | |  | | RAN | |
|  | Reading Tasks |  | Visual-spatial | Verbal |  | Accuracy^a^ | Time^b^ |  | | Letters^b^ | Colours^b^ |
|  |  |  | *(Rho)* | *(Rho)* |  | *(Rho)* | *(Rho)* |  | | *(Rho)* | *(Rho)* |
| TEXT | Accuracy^c^ | ∆_T1_ | 0.09 | -0.01 |  | -0.14 | -0.31 |  | | -0.30 | -0.29 |
|  |  | ∆_T2_ | 0.08 | -0.31 |  | 0.16 | -0.05 |  | | -0.08 | -0.06 |
|  |  | ∆_T3_ | -0.06 | -0.34 |  | -0.09 | -0.04 |  | | -0.09 | -0.30 |
|  | Speed^d^ | ∆_T1_ | 0.17 | -0.20 |  | 0.02 | 0.13 |  | | -0.08 | 0.03 |
|  |  | ∆_T2_ | 0.03 | -0.23 |  | -0.002 | 0.20 |  | | 0.01 | -0.11 |
|  |  | ∆_T3_ | 0.04 | 0.04 |  | 0.05 | -0.09 |  | | -0.20 | -0.29 |
| HF | Accuracy^c^ | ∆_T1_ | -0.09 | -0.22 |  | -0.58** | 0.35 |  | | -0.05 | -0.11 |
|  |  | ∆_T2_ | 0.06 | 0.04 |  | -0.09 | 0.21 |  | | 0.06 | -0.09 |
|  |  | ∆_T3_ | -0.21 | -0.01 |  | -0.14 | -0.13 |  | | -0.16 | -0.24 |
|  | Speed^e^ | ∆_T1_ | -0.38 | -0.38 |  | -0.50* | 0.39 |  | | -0.33 | -0.41 |
|  |  | ∆_T2_ | 0.17 | -0.23 |  | -0.33 | 0.34 |  | | -0.32 | -0.29 |
|  |  | ∆_T3_ | -0.21 | -0.20 |  | -0.19 | -0.03 |  | | -0.27 | -0.40 |
| LF | Accuracy^c^ | ∆_T1_ | -0.30 | -0.19 |  | 0.07 | -0.07 |  | | 0.02 | -0.38 |
|  |  | ∆_T2_ | -0.01 | 0.002 |  | 0.24 | -0.46* |  | | -0.06 | -0.21 |
|  |  | ∆_T3_ | -0.07 | -0.05 |  | 0.24 | -0.16 |  | | -0.01 | -0.31 |
|  | Speed^e^ | ∆_T1_ | -0.27 | -0.29 |  | 0.06 | 0.10 |  | | -0.24 | -0.27 |
|  |  | ∆_T2_ | 0.09 | 0.01 |  | 0.08 | 0.07 | |  | -0.22 | -0.01 |
|  |  | ∆_T3_ | -0.16 | -0.14 |  | 0.36 | 0.15 | |  | 0.06 | -0.32 |
| NW | Accuracy^c^ | ∆_T1_ | 0.05 | 0.28 |  | 0.07 | 0.06 | |  | -0.15 | -0.13 |
|  |  | ∆_T2_ | -0.08 | 0.20 |  | 0.21 | 0.07 | |  | 0.11 | -0.05 |
|  |  | ∆_T3_ | 0.07 | 0.23 |  | 0.30 | -0.14 | |  | 0.05 | 0.02 |
| ^a^ Number of Phonemes; ^b^ Seconds; RAN, Rapid Automatized Naming;  ^c^ Percentage of accuracy, calculated as accuracy/total number of words x 100; ^d^ Seconds/syllables x 100; ^e^ Seconds; HF, High-frequency; LF, Low-frequency; NW, Non-words; ∆_T1_, Changes at T1; ∆_T2_, Changes at T2; ∆_T3_, Changes at T3.  *p ≤ 0.05; **p ≤ 0.01; ^ significant after Bonferroni’s correction (p ≤ 0.0028) | | | | | | | | | | | |

Table S7. Correlations between neuropsychological measures at T0 and ∆_T1_, ∆_T2_, ∆_T3_ for TEXT, HF, LF, and NW reading accuracy and speed, controlling for age, in the sham tDCS condition.

|  |  |  | Sham tDCS  Neuropsychological Tasks at T0 | | | | | | | | |
| --- | --- | --- | --- | --- | --- | --- | --- | --- | --- | --- | --- |
|  |  |  | Working memory_Eff_ | |  | Phoneme Blending | |  | | RAN | |
|  | Reading Tasks |  | Visual-spatial | Verbal |  | Accuracy^a^ | Time^b^ |  | | Letters^b^ | Colours^b^ |
|  |  |  | *(Rho)* | *(Rho)* |  | *(Rho)* | *(Rho)* |  | | *(Rho)* | *(Rho)* |
| TEXT | Accuracy^c^ | ∆_T1_ | 0.29 | 0.29 |  | 0.03 | -0.31 |  | | -0.06 | -0.13 |
|  |  | ∆_T2_ | 0.10 | 0.10 |  | -0.05 | 0.04 |  | | -0.02 | -0.01 |
|  |  | ∆_T3_ | -0.10 | -0.10 |  | -0.38 | 0.23 |  | | 0.13 | 0.04 |
|  | Speed^d^ | ∆_T1_ | -0.25 | -0.25 |  | -0.04 | -0.25 |  | | -0.09 | 0.11 |
|  |  | ∆_T2_ | -0.15 | -0.15 |  | -0.02 | -0.05 |  | | -0.13 | 0.18 |
|  |  | ∆_T3_ | -0.43* | -0.43* |  | -0.20 | 0.15 |  | | 0.08 | 0.39 |
| HF | Accuracy^c^ | ∆_T1_ | -0.07 | -0.07 |  | 0.04 | -0.14 |  | | 0.19 | 0.09 |
|  |  | ∆_T2_ | -0.16 | -0.16 |  | -0.19 | 0.14 |  | | 0.22 | 0.06 |
|  |  | ∆_T3_ | -0.13 | -0.13 |  | -0.08 | -0.26 |  | | 0.11 | -0.17 |
|  | Speed^e^ | ∆_T1_ | 0.02 | 0.02 |  | -0.05 | 0.002 |  | | -0.13 | -0.11 |
|  |  | ∆_T2_ | -0.17 | -0.17 |  | 0.01 | 0.10 |  | | -0.31 | -0.10 |
|  |  | ∆_T3_ | -0.30 | -0.30 |  | -0.05 | 0.12 |  | | -0.05 | 0.27 |
| LF | Accuracy^c^ | ∆_T1_ | 0.16 | 0.16 |  | 0.003 | -0.25 |  | | 0.17 | 0.27 |
|  |  | ∆_T2_ | 0.25 | 0.25 |  | 0.03 | -0.28 |  | | 0.13 | 0.22 |
|  |  | ∆_T3_ | -0.05 | -0.05 |  | -0.13 | -0.06 |  | | 0.45* | 0.22 |
|  | Speed^e^ | ∆_T1_ | -0.24 | -0.24 |  | -0.61** | 0.46* |  | | 0.41* | 0.35 |
|  |  | ∆_T2_ | -0.26 | -0.26 |  | -0.35 | 0.26 | |  | 0.13 | 0.19 |
|  |  | ∆_T3_ | -0.14 | -0.14 |  | -0.34 | 0.14 | |  | 0.17 | 0.27 |
| NW | Accuracy^c^ | ∆_T1_ | -0.16 | -0.16 |  | 0.09 | 0.20 | |  | 0.22 | 0.21 |
|  |  | ∆_T2_ | 0.02 | 0.02 |  | -0.23 | 0.03 | |  | 0.06 | -0.17 |
|  |  | ∆_T3_ | 0.09 | 0.09 |  | 0.001 | -0.20 | |  | 0.36 | 0.24 |
| ^a^ Number of Phonemes; ^b^ Seconds; RAN, Rapid Automatized Naming;  ^c^ Percentage of accuracy, calculated as accuracy/total number of words x 100; ^d^ Seconds/syllables x 100; ^e^ Seconds; HF, High-frequency; LF, Low-frequency; NW, Non-words; ∆_T1_, Changes at T1; ∆_T2_, Changes at T2; ∆_T3_, Changes at T3.  *p ≤ 0.05; **p ≤ 0.01; ^ significant after Bonferroni’s correction (p ≤ 0.0028) | | | | | | | | | | | |

Table S8. Correlations between neuropsychological measures at T0 and NW reading speed at T0, controlling for age, in the active and sham tDCS conditions.

|  |  |  |  |  | |  |  |
| --- | --- | --- | --- | --- | --- | --- | --- |
|  |  | Neuropsychological Tasks |  | Active tDCS | Sham tDCS |  |  |
|  |  |  |  | *(Rho)* | *(Rho)* |  |  |
|  |  | Working memory_Eff_ | Visual-spatial |  |  |  |  |
|  |  |  |  | -0.19 | 0.05 |  |  |
|  |  |  |  |  |  |  |  |
|  |  |  | Verbal |  |  |  |  |
|  |  |  |  | -0.43* | -0.28 |  |  |
|  |  |  |  |  |  |  |  |
|  |  | Phoneme Blending | Accuracy^a^ |  |  |  |  |
|  |  |  |  | 0.04 | -0.36 |  |  |
|  |  |  |  |  |  |  |  |
|  |  |  | Speed^b^ |  |  |  |  |
|  |  |  |  | 0.44* | 0.21 |  |  |
|  |  |  |  |  |  |  |  |
|  |  | RAN | Letters^b^ |  |  |  |  |
|  |  |  |  | 0.44* | 0.41 |  |  |
|  |  |  |  |  |  |  |  |
|  |  |  | Colours^b^ |  |  |  |  |
|  |  |  |  | 0.06 | 0.49 |  |  |
|  |  |  |  |  |  |  |  |

^a^Number of Phonemes; ^b^Seconds; RAN, Rapid Automatized Naming; *p ≤ 0.05; **p ≤ 0.01;
 ^significant after Bonferroni’s correction (p ≤ 0.0083)
